# Classification of Odorants in the Vapor Phase Using Composite Features for a Portable E-Nose System

**DOI:** 10.3390/s121216182

**Published:** 2012-11-22

**Authors:** Sang-Il Choi, Gu-Min Jeong, Chunghoon Kim

**Affiliations:** 1 Department of Applied Computer Engineering, Dankook University, 126 Jukjeon-dong, Suji-gu, Yongin-si, 448-701 Gyeonggi-do, Korea; E-Mail: csichoisi@gmail.com; 2 Electrical Engineering, Kookmin University, 2, 861-1, Jeongeung-dong, Songbuk-gu, 136-702 Seoul, Korea; 3 Korea Institute of Industrial Technology, 1271-18, Sa-3-dong, Sangrok-gu, 426-791 Ansan, Korea; E-Mail: ckim@kitech.re.kr

**Keywords:** e-nose system, vapor classification, composite feature, discriminant features

## Abstract

We present an effective portable e-nose system that performs well even in noisy environments. Considering the characteristics of the e-nose data, we use an image covariance matrix-based method for extracting discriminant features for vapor classification. To construct composite vectors, primitive variables of the data measured by a sensor array are rearranged. Then, composite features are extracted by utilizing the information about the statistical dependency among multiple primitive variables, and a classifier for vapor classification is designed with these composite features. Experimental results with different volatile organic compounds data show that the proposed system has better classification performance than other methods in a noisy environment.

## 1. Introduction

An electronic nose (e-nose) is a device intended to detect and discriminate odorants in the vapor phase [1–6]. While human olfaction sense tends to be easily fatigued, an e-nose has advantages in consistently detecting vapors, including those harmful to the human body. In an early electronic nose, calorimetric sensors were used to perform measurements on vapors, and the measurements were usually expressed in arrays of colors [7]. Such an e-nose system, which was used only in a laboratory environment, utilized complicated analytic procedures, including precise equipment such as gas chromatography (GC) systems or mass spectrometers (MS) combined with sophisticated machine intelligence [8,9]. With recent advances in electrochemical sensors and digital technologies, an e-nose system can support a more portable and intelligent platform for the collection and processing of gas compounds [10].

E-nose systems are used for various purposes, such as in control laboratories for line quality control or production departments, environmental protection [11], the food industry [12], the detection of explosive substances [13], and medical diagnosis in the identification of infections through the examination of odors in breath or tissues [14,15]. Breath analysis has several potential applications in respiratory medicine [16–22]. Also, electronic noses are potentially useful for classifying and subphenotyping of patients with different respiratory diseases [23].

Most e-nose systems consist of a nonspecific sensor array and a computing system [24]. Each sensor in a sensor array reacts to volatile compounds on contact. The adsorption of volatile compounds on the sensor surface causes a physical change of the sensor. A specific response is recorded by the electronic interface transforming the signal into the numerical data in vector form. In a computing system, various pattern recognition techniques, such as feature extraction or feature selection methods, can be used to classify the data into a suitable class. Some methods extract the discriminant features for classification by using LDA (Linear Discriminant Analysis) [25] or combine Fisher discriminant analysis with modified Sammon mapping [26]. In work [27,28], a vector machine such as the support vector machine or relevance vector machine was used to classify vapors. In work [10], after refining the e-nose data through the feature feedback process [29], vapor classification was performed by using LDA and a nearest neighbor classifier.

Various feature extraction methods have different characteristics depending on the problems to be solved. For example, PCA (Principal Component Analysis) [30] does not utilize the class information of the data but finds the projection vectors from the data sample that minimize the mean square error of approximating the data. Thus, PCA is more appropriate for a data representation problem rather than a classification problem. On the other hand, LDA [31] finds a linear transformation that maximizes the ratio of the between-class scatter matrix (*S_B_*) and the within-class scatter matrix (*S_W_*). Since LDA assumes that the samples in each class are normally distributed, it performs well for data that satisfies this assumption. In the case of detection problems such as one-class classification problems, BDA (Biased Discriminant Analysis) [32] performs well using the scatter matrix of the positive (*S_P_*) and negative (*S_N_*) samples. Thus, it is important to determine an appropriate method depending on the properties of the data, which has a great effect on the final classification results.

**Figure 1.**
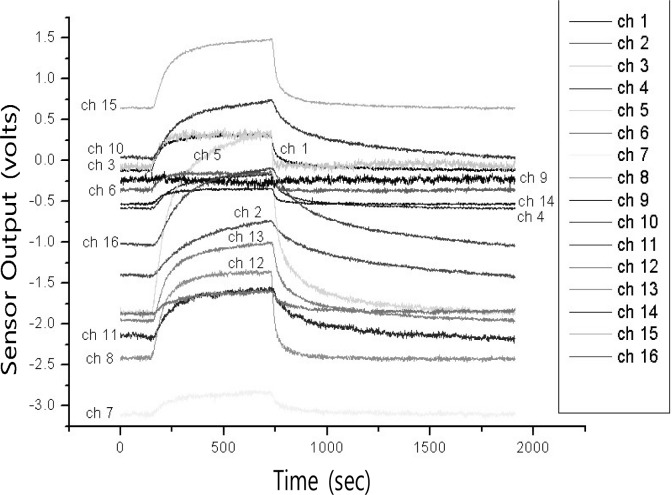
Typical time-responses of 16 channel sensor array with respect to inflow of acetone vapor at 5,000 ppm [10].

In this paper, we present an e-nose system robust to noisy environments by using composite features for vapor classification. The e-nose sensor used measures vapors with a speed of 10 Hz, which corresponds to a sampling rate of 2,000 points per 200 seconds [7]. Since a sensor array has 16 channels, each measured data sample contains 32,000 primitive variables, which is likely to result in computational burden. Figure 1 shows one of the typical time responses of a 16-channel sensor array with respect to the inflow of acetone vapor [10]. As can be seen in Figure 1, there is a strong correlation between adjacent time responses in a sample. This makes it plausible to use a method that is suitable for high-dimensional data and utilizes information on statistical dependency among multiple primitive variables.

There are several methods such as 2DFLD (Two-Dimensional Fisher Linear Discriminant) [33], MatFLDA (Matrixized Fisher Linear Discriminant Analysis) [34] and C-LDA (Composite LDA) [35], in which image covariance matrices were used instead of covariance matrices. Each element of an image covariance matrix is defined as the inner product of two composite vectors, each of which is obtained from a predefined window in a data sample [36]. 2DLDA and MatFLDA can be viewed as a particular type of C-LDA because C-LDA becomes identical to these forms when the composite vector is defined as a row or column vector [37]. The composite features, which are used to construct a classifier, are obtained by linear combinations of the composite vectors. Kim *et al*. [36] showed that composite features are effective for data that has a large correlation between primitive variables or high-dimensional data such as face images. Therefore, we expect that classification using composite features performs very effectively for e-nose data. Moreover, the size of an image covariance matrix can be controlled by changing the window size or by overlapping the windows. This is another great advantage in the classification of high-dimensional data such as e-nose data, because manipulation of a large covariance matrix can be avoided and consequently the SSS (Small Sample Size) problem [38] can be solved. By designing a classifier for vapor classification with composite features, we obtained very good results even in a noisy environment. Experimental results show that our system is very effective in vapor classification in terms of not only classification rates but also robustness to noise.

The rest of this paper is organized as follows. Section 2 introduces an image covariance matrix and presents the derivation of C-LDA. Section 3 explains how e-nose data that are measured by a sensor array are represented using composite vectors and how composite features are extracted for vapor classification. Section 4 describes the experimental results and the conclusion follows in Section 5.

## 2. Composite Feature Extraction Based on Image Covariance Matrix

In pattern recognition, data is generally stored in vectors, whose elements are called primitive variables [36]. Conventional feature extraction methods such as PCA, LDA, or BDA use the covariance of primitive variables. In each method, the features are extracted by solving the particular objective function, which is defined using various types of covariance matrices. However, when dealing with high-dimensional data, a huge number of combinations should be computed for obtaining the covariance matrix. Since there are high correlations between neighboring primitive variables, it is redundant to use all of these combinations. Moreover, it is likely to encounter the SSS problem in the process of eigenvalue analysis of the covariance matrix.

**Figure 2.**
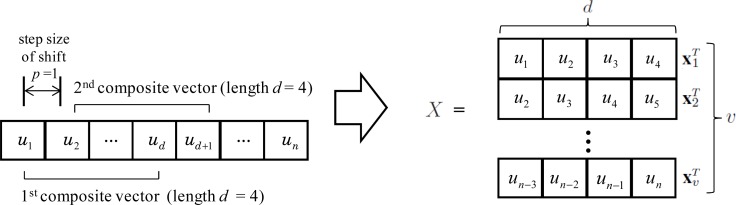
Example of constructing composite vectors (*d* = 4 and *p* = 1).

In the feature extraction methods based on image covariance matrix, the covariance is calculated from two sets of primitive variables instead of two primitive variables. Each sets of primitive variables is called a “composite vector”. Let *U* denote a set of *n* primitive variables {*u*_1_, *u*_2_, .., *u_n_*}. Then, a composite vector **x***_i_* ∈ ℝ*^d^* consists of *d*(*< n*) primitive variables that are come from a predefined window in a data sample (Figure 2). If shifting a window as much as *p*, the number of composite vectors *v* is $\left\lfloor \frac{n-d}{p}\right\rfloor +1$, where ⌊·⌋ is the floor operator that gives the largest integer value no greater than the value inside the operator. Depending on the length of the window *d* and the step size of shift *p*, the dimension and number of composite vectors are determined.

Let *X*(*k*) = [**x**_1_(*k*)**x**_2_(*k*)..**x***_v_*(*k*)]*^T^* ∈ ℝ*^v×d^* be a set of composite vectors obtained from the *k*-th data sample. In Figure 2, *d* and *p* are set to 4 and 1, respectively. Each element of an image covariance matrix *c_ij_* can be obtained from the inner product of two composite vectors and is defined as

(1) $$c_{\mathrm{ij}}=E[{\left(x_{i}-{\bar{x}}_{i}\right)}^{T}(x_{j}-{\bar{x}}_{j})],\;\;\;\;\;\;\;i,j=1,2,..,v$$

where **x̄***_i_* and **x̄***_j_* are the mean vectors of **x***_i_* and **x***_j_*, respectively. Since *c_ij_* corresponds to the sum of the covariances between the corresponding primitive variables, it contains information on statistical dependency among multiple primitive variables. Moreover, when using image covariances obtained from composite vectors, the size of the image covariance matrix can be reduced greatly, which enables us to avoid manipulation of large covariance matrices and to solve the SSS problem.

When the training set contains *N* samples and *c* classes, each of which has *N_i_* samples, between (*C_B_*)- and within(*C_W_*)-class covariance matrices are defined as

(2) $$\begin{array}{l} C_{B}=\sum_{i=1}^{c}\frac{N_{i}}{N}(M_{i}-M){\left(M_{i}-M\right)}^{T}\\ C_{W}=\frac{1}{N}\sum_{i=1}^{c}\sum_{X(k)\in c_{i}}(X(k)-M_{i}){\left(X(k)-M_{i}\right)}^{T} \end{array}$$

where, *M* and *M_i_* are the mean of the whole training samples and the mean of the class *c_i_*, respectively. Then, the objective function of C-LDA is defined as follows:

(3) $$W_{\mathrm{Com}}=\text{arg}\underset{W}{\text{max}}\frac{\left|W^{T}C_{B}W\right|}{\left|W^{T}C_{W}W\right|}$$

The projection matrix *W_Com_* ∈ ℝ*^v×m^* consists of projection vectors **w***_i_*(= [*w_i_*_1_*, .., w_iv_*]*^T^*)s (*W_Com_* = [**w**_1_**w**_2_..**w***_m_*]). The set of composite features *Y* (*k*) is obtained from *X*(*k*) as

(4) $$Y(k)={\left(W_{\mathrm{Com}}\right)}^{T}X(k),\;\;\;\;\;\;\;k=1,2,\ldots ,N$$

*Y* (*k*) ∈ ℝ*^m×d^* has *m* composite features [**y**_1_(*k*) . . . **y***_m_*(*k*)]*^T^*, and each composite feature y*_i_*(*k*) is a *d*-dimensional vector. These composite features are used for classification.

## 3. Vapor Classification Using Composite Features

### 3.1. Experimental Setup for the Acquisition of E-Nose Data

The sensor array was implemented by dispensing the CB polymer composite-solvent solution in the micromachined gas sensor array chip in [7]. It consists of 16 separate sensors with an interdigitated electrode, microheater, and micromachined membrane in each channel for further temperature-controlled measurement applications. The resistance change of each polymer composite film was monitored in response to the incorporation of chemical vapor. The resistance change of polymer composite film was amplified by 20 times and recorded every 0.1 s. Measurement was performed after the sensor array was placed into the chamber and the signal of resistance was stabilized. Each measurement consisted of three steps of stabilization (30 s), exposure (60 s), and purge (90 s) [39]. The measured data are collected in PC using data acquisition (DAQ) board DAQ6062E and LabVIEW (National Instrumentation, USA). The voltage-divider operated in the range from −10 V to +10 V and gains of 16 identical amplifiers were set to 10 (output/input voltage) for maximum DAQ resolution [7].

### 3.2. Vapor Classification Using Composite Features

**Figure 3.**
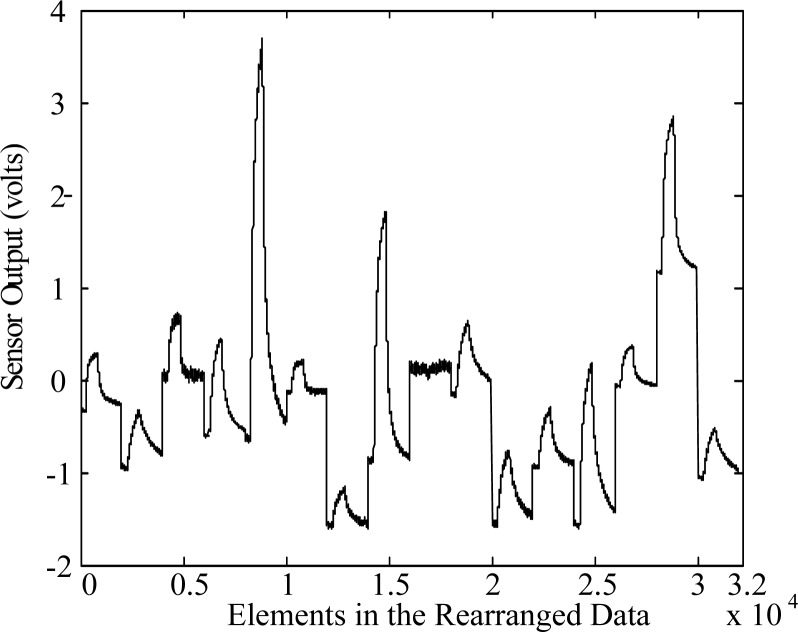
Data sample that is transformed into a 32,000 dimensional vector form.

Now, we design a vapor classification system using composite features. The VOC (Volatile Organic Compounds) measurement data used consists of 8 classes, which are acetone, benzene, cyclohexane, ethanol, heptane, methanol, propanol and toluene [7]. The data set contains 160 samples, *i.e.*, *N* = 160. Each sample was measured through 16 channels over 2,000 time points, which can be viewed as a 16 × 2, 000 matrix. In order to make composite vectors effectively, we transform this matrix into a 32,000-dimensional vector using a lexicographic ordering operator [35]. Figure 3 shows one example of the data that is transformed into a vector form. Then, we construct a composite vector by grouping adjacent elements and moving as much as the step size of shift.

**Figure 4.**
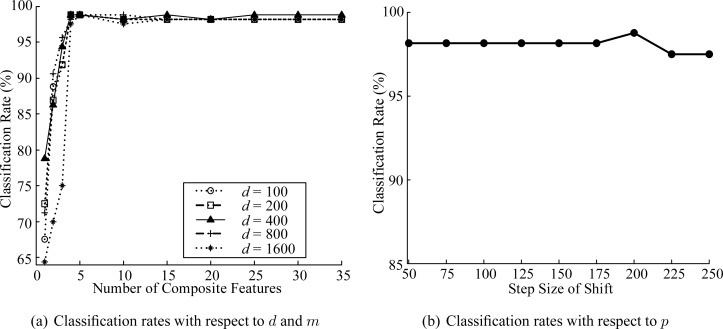
Classification rates for various parameters.

The length of the window (*d*), the number of composite features (*m*) and the step size of shift (*p*) are important parameters that influence the classification performance. We investigated the classification rates with respect to *d*, *m*, and *p*. Figure 4(a) shows the classification rates with respect to *d* and *m*. In this case, we set *p* = *d/*2 as in [35]. As can be seen in the figure, the classification rates are not sensitive to *d* if *m* is properly decided. We set *d* and *m* to 400 and 25, respectively. Then, we investigated the classification rates with respect to *p*. As can be seen in Figure 4(b), the classification rates are not sensitive to *p* and the classification rate of *p* = 200 was slightly better than those of other *p* values. Therefore, we set *p* to 200.

**Figure 5.**
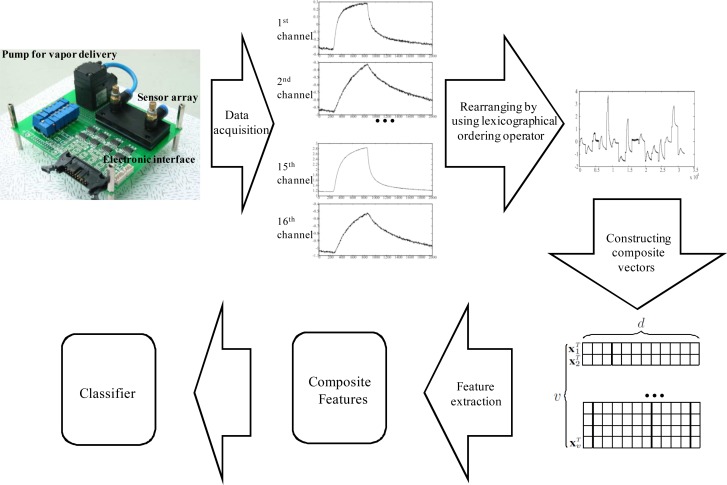
Overall procedure of the e-nose system.

The set of extracted composite features consists of *m* vectors of dimension *d*, and we need to define the distance metrics in this subspace. The Euclidean (*L*2) distance between *Y* (*i*) = [**y**_1_(*i*) . . . **y***_m_*(*i*)]*^T^* and *Y* (*j*) = [**y**_1_(*j*) . . . **y***_m_*(*j*)]*^T^* are defined as

(5) $$d_{L2}(Y(i),Y(j))={\left\{\sum_{t=1}^{m}{\left\|y_{t}(i)-y_{t}(j)\right\|}^{2}\right\}}^{1/2}$$

where ‖ · ‖ is the 2-norm. The distance between **y***_t_*(*i*) and **y***_t_*(*j*) is obtained from the Euclidean distance in the *d*-dimensional space. The *L*2 distance is calculated by taking the square root of the squared sum of these distances. For classification, one nearest neighbor classifier [38] is used based on the *L*2 distance. The overall procedure of our system is shown in Figure 5.

## 4. Experimental Results

In order to evaluate the classification rates, we performed 8-fold cross validation [40] 8 times and computed the average classification rate. In this scheme, one sample from each class was randomly selected for testing, while the remaining samples were used for training. There were 140 data samples in the training set and 20 samples for testing. Each data sample in the training set was also normalized using the mean and the variance of the training set.

**Figure 6.**
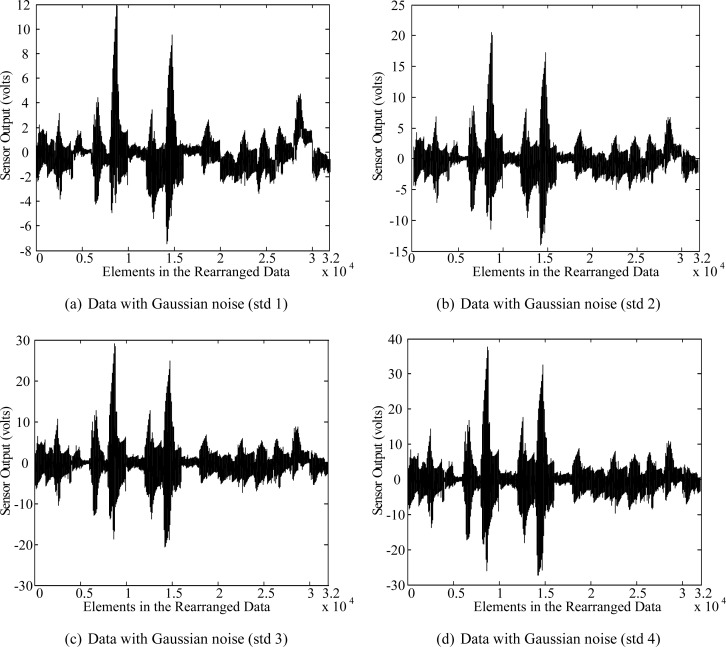
E-Nose data with Gaussian noise of standard deviation 1–4.

**Figure 7.**
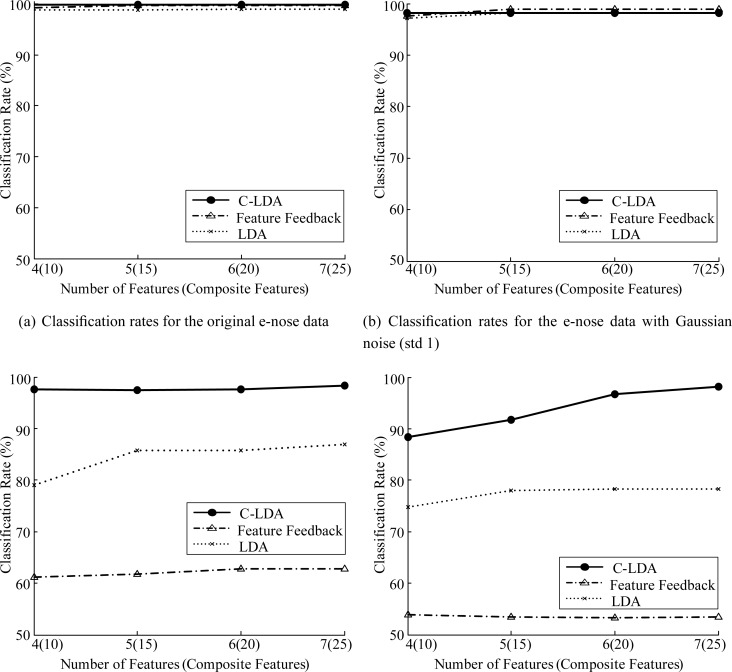
Classification rates for the e-nose data.

We compared the classification performance of the C-LDA method with those of the LDA method and the feature feedback method (FF) [10]. In addition, in order to see the robustness of each method to the noise, which is likely to occur in sensing data, we added Gaussian noise with standard deviation from 1 to 4 to each data sample. Figures 6 and 7 show some examples of the data with Gaussian noise and classification rates of each case, respectively.

As can be seen in Figure 7, all the methods give high classification rates for the original data. However, as the degree of noise increases, the classification rates of LDA and FF decrease rapidly. In contrast, C-LDA gives consistently high classification rates of 95.0% ∼ 98.1%, which shows that our system performs reliably in a noisy environment. In Figure 7(c), the classification rates of C-LDA are 11.1% and 35.2% higher than that of LDA and FF, respectively. In the case of Figure 7(d), C-LDA gives 16.6% and 41.1% higher classification rates than LDA and FF, respectively.

## 5. Conclusions

We presented a reliable e-nose system using an appropriate feature extraction method based on the characteristics of e-nose data. C-LDA is a general method that uses the image covariance matrix, which is a covariance matrix of composite vectors, instead of the covariance of primitive variables. Since the adjacent primitive variables are strongly correlated in e-nose data, the proposed method showed better performance than other methods. In addition, we can avoid the SSS problem, which occurs in dealing with high-dimensional data such as e-nose data, by using the small-sized image covariance matrix, instead of large-sized covariance matrix. By extracting composite features after rearranging the primitive variables of e-nose data samples, we utilized information about the statistical dependency among the multiple primitive variables of the e-nose data. By investigating classification rates for the various lengths of composite vectors and the step sizes of shift, we found an adequate parameter set for vapor classification. In a real environment, the data measured by the portable e-nose system is likely to be corrupted by noise, which interferes with extracting good features for classification. Through experimental results, we showed that the proposed system gave good classification performance even in a noisy environment.
